# The combined effects of elevated predation risk and anthropogenic noise on dwarf mongoose vigilance behaviour

**DOI:** 10.1098/rsbl.2024.0645

**Published:** 2025-02-12

**Authors:** Lucy Westover, Amy Morris-Drake, Megan Layton, Julie M. Kern, Josh J. Arbon, Andrew N. Radford

**Affiliations:** ^1^ School of Biological Sciences, University of Bristol, 24 Tyndall Avenue, Bristol BS8 1TQ, UK

**Keywords:** anthropogenic noise, predation risk, dual stressors, playback experiment, vigilance, risk-disturbance hypothesis

## Abstract

Anthropogenic noise is a pervasive pollutant in the world’s ecosystems, with numerous studies demonstrating negative physiological, developmental and behavioural impacts across taxa. However, research has tended to focus on anthropogenic noise in isolation; many species often experience this pollutant in conjunction with other anthropogenic and natural stressors. Here, we used a field experiment to investigate the combined effects of a sequential elevation in perceived predation risk followed by exposure to road noise on the vigilance behaviour of dwarf mongooses (*Helogale parvula*). As expected, both alarm-call playback (simulating a greater predation risk) and road-noise playback independently led to more vigilance compared to close-call and ambient-sound (control) playbacks, respectively. The two stressors had an equivalent effect on total vigilance, lending support to the risk-disturbance hypothesis. The combination of the two stressors did not, however, generate a significantly different amount of vigilance compared to road-noise playback alone. Thus, our experiment provides further evidence that anthropogenic noise can influence the vigilance–foraging trade-off but no indication of an additive or synergistic effect when combined with the natural stressor of elevated predation risk. Further investigation of combined-stressor effects is critical if we are to understand the true impacts of anthropogenic disturbances on species and communities.

## 1. Introduction

Anthropogenic (man-made) noise is a pervasive pollutant in the world’s terrestrial and marine ecosystems due to human population growth, urbanization, resource extraction and transportation [1,2]. In the last 20 years, research has demonstrated negative physiological, behavioural, developmental and fitness consequences of noise for a wide range of non-human animals [1,3–5]. Anthropogenic noise can cause behavioural changes by masking acoustic signals or cues [6], distracting individuals [7], acting as a stressor [8] and by being perceived as a threat [9]. Masking of acoustic signals can change how both senders and receivers behave [10,11], and masking can also obscure important acoustic cues from other animals and the environment [12,13]. Noise can divert an individual’s finite attention from their primary task and thus detrimentally impact, for instance, foraging and anti-predator behaviour [7,14,15]. Noise can induce physiological stress, which can, in turn, lead to maladaptive behavioural responses [8,16]. The perception of noise as a threat can cause behavioural changes, including increased vigilance and reduced foraging, that are qualitatively similar to those seen in a predation context [17]; this is known as the risk-disturbance hypothesis [18]. Most behavioural studies on wild animals have documented the effects of anthropogenic noise in isolation, despite it often occurring with other anthropogenic and natural stressors; a greater understanding of the combined effects of multiple stressors is needed to determine the true effects of human disturbance [1,19].

Multiple stressors can potentially act additively (the combined effect is the sum of the individual stressor effects), antagonistically (the combined effect is less than the sum of the individual stressor effects) or synergistically (the combined effect is greater than the sum of the individual stressor effects) [19–21]. Relevant research has mostly focused on combinations of anthropogenic stressors, especially noise and light pollution [19,22–24]. For example, Caribbean hermit crabs (*Coenobita clypeatus*) exposed to both noise and light pollution allowed a predator to move even closer compared to when there was just road-noise exposure, indicating an additive effect [7,22]. When artificial light exposure was combined with noise pollution, a synergistic effect was found on bird abundance: species that decreased in abundance due to noise exposure showed even greater declines with the introduction of light pollution [25]. However, animals face not only multiple anthropogenic stressors but also experience natural stressors like predation risk. Some research has shown that anthropogenic disturbances can cause a more extreme response than predation risk. For instance, European mink (*Mustela lutreola*) hid when presented with predator faecal odours but showed a more exaggerated hiding response when exposed to anthropogenic noise [26]. In common lesser escuerzos (*Odontophrynus americanus*), road noise and conspecific chorus noise in combination caused a greater increase in call dominant frequency than either stressor individually [27]. Contrastingly, chipmunks (*Tamias striatus*) and white-footed mice (*Peromyscus leucopus*) reduced their food intake significantly when exposed to predation risk, but simultaneous exposure to road noise seemed to eliminate this effect [28]. More experimental tests comparing animal responses to anthropogenic noise and natural stressors (such as predation risk), as well as their combined effect, are needed to understand fully the impacts of this global pollutant.

We used a field experiment to investigate the individual and combined effects of elevated predation risk and road noise on vigilance behaviour in wild dwarf mongooses (*Helogale parvula*). Dwarf mongooses are diurnal, cooperative breeders living in groups of up to 30 individuals [29,30]. Foragers spend most of the time digging with their heads down, so they are unable to be vigilant simultaneously; group members warn others of danger using various alarm calls [31]. Road noise has a range of effects on dwarf mongoose behaviour: they respond less to surveillance calls of sentinels (raised guards), increase their vigilance, respond less appropriately to olfactory predator cues and are less likely to flee when hearing heterospecific alarm calls [30,32–34]. To elevate the perceived predation risk, we used alarm-call playback in contrast to playback of close calls (vocalizations given whilst foraging that are unrelated to predation risk). Following the initial alarm- or close-call playback, individuals received a subsequent playback of either ambient sound or road noise. We adopted this sequential, rather than simultaneous, playback methodology because we were interested in a combined stressor effect rather than the potential for noise to mask acoustic information. Based on the risk-disturbance hypothesis, we predicted that road-noise playback would result in greater vigilance than ambient-sound playback and that the two stressors (elevated predation risk and road-noise playback) would individually cause an equivalent increase in vigilance. If the combination of elevated predation risk and anthropogenic noise had an additive or synergistic effect, we expected a further increase in vigilance in response to road-noise playback that followed alarm-call playback. Contrastingly, if these stressors acted antagonistically, we expected to see less vigilance behaviour following sequential alarm-call and road-noise playbacks than the sum of the effects seen from the two stressors individually.

## 2. Methods

### (a) Study site and species

We collected data in July–September 2021 on Sorabi Rock Lodge Reserve, South Africa, the site of the Dwarf Mongoose Research Project (DMRP); full details of the study site are provided elsewhere [35]. There is a main road (the R530) running alongside the reserve, with sporadic passing traffic, making road noise a relevant local pollutant. All focal groups live within 2 km of the road, and traffic noise can be heard throughout the reserve. Dwarf mongoose groups comprise a dominant breeding pair and subordinate adult helpers of both sexes [29]. Group members move around their territory together, foraging for insects and small vertebrates; foragers emit low-amplitude ‘close’ calls continuously [36]. Individuals produce specific alarm calls to warn groupmates about the presence of aerial or terrestrial predators [31]. We ran our playback experiment on adults (individuals older than 1 year) of both sexes from six habituated wild groups (mean ± s.d. group size = 11.7 ± 5.2, range = 6−20). Further details in the electronic supplementary material.

### (b) Playback experiment

We used a repeated-measures experimental design whereby each focal individual (*n* = 17) received four treatments: alarm call then road noise, alarm call then ambient sound, close calls then road noise and close calls then ambient sound. We made sound recordings in calm weather conditions using a solid-state recorder and shotgun microphone (full details in the electronic supplementary material). Ambient-sound recordings were made in the centre of each group’s territory at least 50 m from any mongoose or human activity. They were collected around midday to ensure conditions were relevant to both morning and afternoon experimental sessions. Road-noise recordings were made 10 m from the R530 road, with the microphone facing the passing traffic. Matched close calls and aerial alarm calls were recorded from the same subordinate group members; recorded alarm calls were given in response to natural predators (see electronic supplementary material).

We created all playback tracks in Audacity (v. 3.0.2). We made unique 2-min ambient-sound and road-noise tracks for each focal individual. Road-noise tracks contained the mean number and type of vehicles observed during traffic counts on the R530 [30,34]. Close-call tracks comprised 2 s of ambient sound, then four calls each separated by 12 s of ambient sound, and a final 2 s of ambient sound; ambient sound was from the territory of the playback individual. Alarm-call tracks were identical to close-call tracks except that the final call was an aerial alarm call. Each focal individual was played close calls and alarm calls of the same groupmate, but different focal individuals in the same group received playbacks from different groupmates. A sound meter was used to ensure that playback levels were ecologically relevant and standardized across all tracks of the same type. Full details of track creation and playback are in the electronic supplementary material.

We carried out all four trials to a focal individual within 5 days, with group size the same for all trials. Each individual received all its trials either in the morning (07.30−12.00) or the afternoon (12.30−17.00). There was a maximum of two trials per day; at least 30 min was left between trials on the same day. Treatment order was counterbalanced between focal individuals. We only carried out trials when a standardized set of social and environmental conditions were met (electronic supplementary material). Following the close- or alarm-call playback, there was a 1 min observation period. We chose this 1 min observation period to be long enough to observe any difference in behaviour resulting from either the close- or alarm-call playback while minimizing the risk of the trial needing to be abandoned. We then played the focal individual either a road-noise or ambient-sound track, during which there was a second 1 min observation period (to match the duration of the first observation period) that began with playback initiation. All vigilance behaviour (head up and scanning the area) by individuals remaining on the ground was recorded continuously into a Dictaphone (Sony ICD-PX370) during both observation periods; very occasional occurrences of sentinel behaviour were not included in analyses.

### (c) Data analysis

All statistical analyses were conducted in R v. 4.4.0 [37]. We ran linear mixed models (LMMs) and generalized linear mixed models (GLMMs) using the lmer/glmer functions in the ‘lme4’ package [38] to control for the matched experimental design and the use of more than one focal individual per group. All models had Individual ID nested within Group ID fitted as random effects; individuals were always present in the same groups during the experiment. We applied a weak Bayesian prior to the random-effects structure of all models using the blmer()/bglmer() functions from the ‘blme’ package [39]. These act as wrapper functions for lme4 models, fitting a weakly informative prior to the covariance matrix (here a Wishart distribution), helping to avoid singular model fit. Model fit and assumptions were checked using diagnostic functions from the ‘DHARMa’ package [40]. Model simplification to generate a final model entailed the removal of non-significant interaction terms using an alpha of 0.05; main effects selected *a priori* were always retained. Fixed factor estimates and confidence intervals were generated from the final model; *p*-values were calculated via likelihood ratio tests (LRTs) between the final model and the final model minus the term of interest. For pairwise comparisons between multi-level factors, we conducted *post-hoc* Tukey tests using the lsmeans() function from the ‘emmeans’ package [41].

We used alarm-call playback to simulate an increased predation risk, so we first assessed whether that elicited more vigilance than close-call playback. Due to the low levels of vigilance seen following close-call playbacks, we converted the data into a binary response (whether or not any vigilance was seen in the minute following playback). This dataset was therefore analysed using a GLMM with a binomial error structure and logit link function (model 1). We included call type (close- and alarm-call), trial order (first, second, third and fourth) and their interaction as fixed effects. As intended, mongooses were more likely to be vigilant following an alarm-call playback compared with a close-call playback (LRT: χ^2^_1_ = 36.592, *p*<0.001; electronic supplementary material, table S1).

For the two main sets of analyses, we generated three response variables. We initially assessed the total time spent vigilant for the relevant period and then broke vigilance into its two key components: the number of separate vigilance occurrences and the mean duration of these bouts. First, we asked how vigilance was affected by the two separate stressors (models 2 a–c), comparing behaviour in the 1 min period during road-noise playback (using the trial where there was initial close-call playback) with the 1 min period following an alarm-call playback (using the two relevant trials and taking the mean for total vigilance time and mean bout duration, so that there was the same number of datapoints per stressor). We included stressor (road noise and alarm call) as a fixed effect; as mean values were used for alarm-call responses, trial order was not included. We then asked how vigilance was affected by the combination of alarm-call and road-noise playback versus one or neither stressor, comparing the first minute of either the ambient sound or road-noise playback in all four trials. One outlier was removed for these analyses. We included treatment (close call then ambient sound, close call then road noise, alarm call then ambient sound and alarm call then road noise), trial order (first, second, third and fourth) and their interaction as fixed effects. Models assessing the total time spent vigilant and mean duration of vigilance bouts (models 2 a,c, 3 a,c) were fitted as LMMs with square-root transformed data, whilst both models assessing the number of vigilance occurrences (models 2b, 3b) were fitted as GLMMs with a Poisson error structure and log link function.

## 3. Results

**Figure 1.**
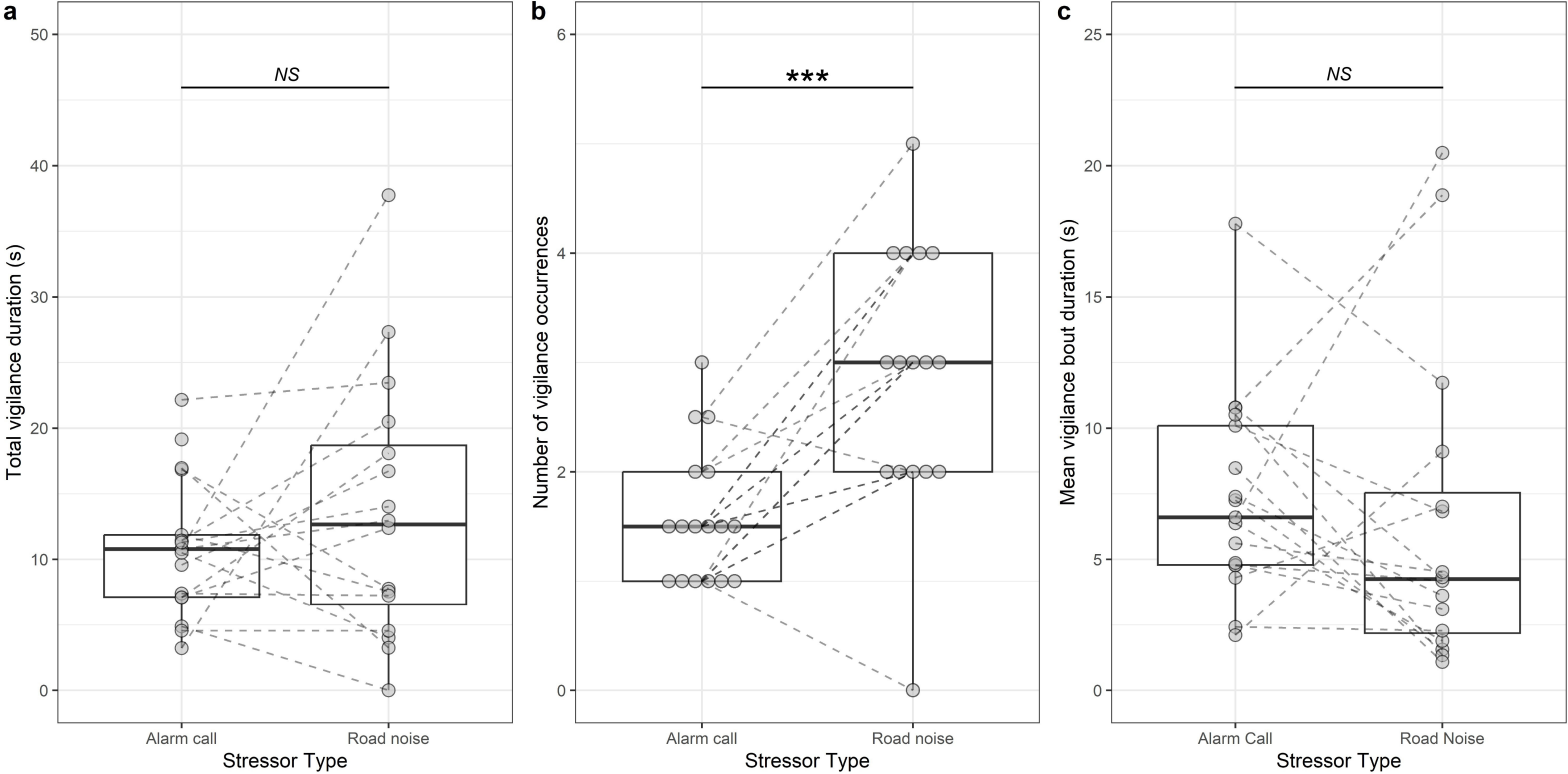
The effect of alarm-call and road-noise playbacks on (*a*) total vigilance duration, (*b*) number of vigilance occurrences and (*c*) mean vigilance bout duration. Boxplots show median and interquartile ranges (IQR), arms 1.5 × IQR. Raw data points (dots) from the same focal individual are connected by dashed lines. In (*b*), points are spaced to allow easier assessment of the number at each value. There can be multiple data points of the same value, and so the number of visible lines is less than the sample size (*n* = 17 individuals in six groups). ***p < 0.001, NS = non-significant.

Overall, there was no significant difference in the time that individuals spent vigilant in response to road-noise playback compared with alarm-call playback (χ^2^_1_ = 0.820, *p* = 0.365; electronic supplementary material, table S2a; figure 1*a*). However, individuals conducted an estimated 1.8 times more vigilance bouts (95% CI: 1.2x−2.8x) when hearing road noise compared to immediately after an alarm call (χ^2^_1_ = 8.208, *p* = 0.004; electronic supplementary material, table S2b; figure 1*b*). In contrast, the mean duration of vigilance bouts was not significantly different after hearing an alarm call rather than during road-noise playback (χ^2^_1_ = 1.378, *p* = 0.240; electronic supplementary material, table S2c; figure 1*c*).

**Figure 2.**
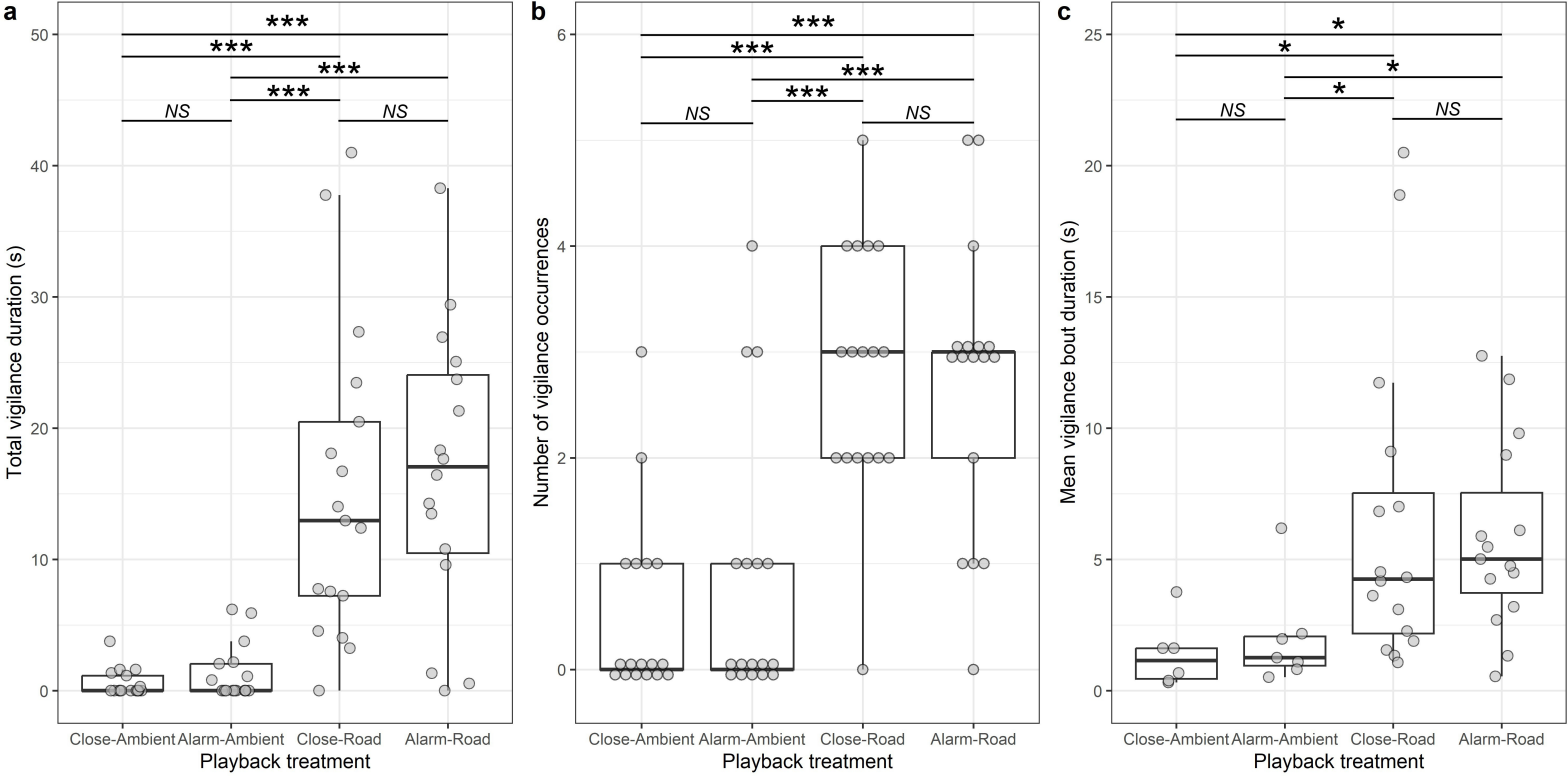
The effect of the four different treatments on (*a*) total vigilance duration, (*b*) number of vigilance occurrences and (*c*) mean vigilance bout duration. Boxplots show median and interquartile ranges (IQR), arms 1.5 × IQR; raw data points shown as dots. In (a) and (c), points are jittered, and in (b) points are spaced, to allow easier assessment of the number at each value. *n* = 17 individuals in six groups. *p < 0.05, ***p < 0.001, NS = non-significant.

The total time that an individual spent vigilant in response to the two consecutive playbacks was affected by treatment (LRT: χ^2^_3_ = 61.050, *p* < 0.001; electronic supplementary material, table S3a). Overall, road-noise playback elicited 15.0 s (95% CI: 10.6−19.3 s) more vigilance than ambient-sound playback during the 1 min observation period, but there was no significant difference found between the two road-noise treatments; hearing an alarm call rather than close calls prior to the road-noise playback did not lead to individuals spending more time being vigilant (table 1a; figure 2*a*). The treatment effect on total vigilance time was driven by differences in both the number of vigilance occurrences (χ^2^_3_ = 48.942, *p* < 0.001; electronic supplementary material, table S3b) and their mean duration (χ^2^_3_ = 14.603, *p* = 0.002; electronic supplementary material, table S3c). Mongooses exhibited 2.1 (95% CI: 1.4−2.9) more vigilance occurrences (figure 2*b*), with vigilance bouts being 4.4 s (95% CI: 1.8−7.0 s) longer (figure 2*c*), in the 1 min period during road-noise playbacks compared to ambient-sound playbacks. However, there was no significant difference in either measure depending on whether alarm-call or close-call playback preceded the road noise (table 1b,c; figure 2*b*,*c*).

**Table 1.** *Post*-*hoc* Tukey test output investigating the effects of the different treatments on the three vigilance response variables. Estimates (Est) and s.e. are shown on the log scale and are for the bold treatment relative to the non-bold treatment indicated in the ‘*post-hoc* comparison’ column. *n* = 17 individuals in six groups. Kenward–Roger degrees of freedom are given where relevant.

*post-hoc* comparison	(a) total vigilance duration			(b) number of vigilance occurrences			(c) mean vigilance bout duration		
	Est (s.e.)	*t* (d.f.)	*p*	Est (s.e.)	*z*	*p*	Est (s.e.)	*t* (d.f.)	*p*
close–ambient **alarm–ambient**	0.254 (0.421)	0.604 (46)	0.930	0.441 (0.427)	1.033	0.730	0.135 (0.458)	0.295 (30.6)	0.991
close–ambient **close–road**	3.131 (0.421)	7.165 (46)	<0.001	1.675 (0.363)	4.610	<0.001	1.207 (0.403)	2.996 (30.6)	0.026
close–ambient **alarm–road**	3.332 (0.430)	8.061 (46.5)	<0.001	1.690 (0.366)	4.615	<0.001	1.254 (0.409)	3.068 (29.9)	0.022
alarm–ambient **close–road**	2.876 (0.421)	6.581 (46)	<0.001	1.234 (0.304)	4.060	<0.001	1.072 (0.370)	2.894 (28.2)	0.035
alarm–ambient **alarm–road**	3.078 (0.431)	7.467 (46.5)	<0.001	1.249 (0.307)	4.065	<0.001	1.119 (0.381)	2.939 (28.9)	0.031
close–road **alarm–road**	0.201 (0.430)	0.916 (46.5)	0.966	0.016 (0.207)	0.074	0.999	0.047 (0.280)	0.169 (24.7)	0.998

## 4. Discussion

Both alarm-call playback (simulating an elevated predation risk) and road-noise playback independently led to more vigilance by dwarf mongoose foragers than control playbacks of close calls and ambient sound, respectively. There was some limited evidence that road noise and elevated predation risk had different influences on vigilance behaviour. Vigilance was not greater in the dual-stressor treatment (road noise preceded by alarm call) than when an individual was exposed to road noise alone (i.e. following close calls). Thus, our field experiment supported the risk-disturbance hypothesis in relation to anthropogenic noise but provided no evidence for an additive or synergistic effect of the combined stressors.

As predicted, alarm-call playback led to greater vigilance, which is consistent with previous work on dwarf mongooses [31] and various other species [42–44]. Greater vigilance is expected when the perceived predation risk is higher, as this allows additional information gathering about potential danger. Also, as predicted, road-noise playback led to more vigilant behaviour, as previously reported in dwarf mongooses [34] and other species [9,28,45]. This effect of road noise could be due to the potential masking of important acoustic cues (e.g. those generated by predators or heterospecific alarm calls) and signals (e.g. conspecific alarm calls) that indicate danger [32,46], so that the mongooses compensate visually with increased vigilance [6]. Alternatively, individuals may be perceiving the road noise as a threat in the same way that they would a predation risk [47,48].

Our direct comparison of the response to alarm-call and road-noise playback found no significant difference in the overall time spent vigilant. However, there was some evidence that the two stressors affected vigilance behaviour differently in a more nuanced way: dwarf mongooses looked up more often when exposed to road noise compared to when there was an increased predation risk; in contrast, vigilance bouts were, on average, shorter in response to road-noise playback, although this difference with alarm-call playback was not statistically significant. It is possible that vigilance bouts were triggered by separate vehicle passes in the road-noise recording (there were more of these than alarm-call events in that playback), but that vigilance ceased relatively quickly each time once the individual realized that the sound did not represent a threat. The similar overall vigilance response to road noise and alarm-call playbacks lends support to the risk-disturbance hypothesis [18] whereby animals react in the same way that they do to natural predators when they are exposed to anthropogenic disturbance. To date, few studies have explored this directly, and the studies that have been done have conflicting results. For example, work on pygmy marmosets (*Cebuella pygmaea*) found that while noise could change the behaviour of these animals, there was little evidence to support the risk-disturbance hypothesis [49]. However, Carolina chickadees (*Poecile carolinensis*) and tufted titmice (*Baeolophus bicolor*) responded to traffic noise in the same way that they would in the presence of a predator by reducing the distance between nearest neighbours [50]. Teasing apart the exact mechanism of noise effect (i.e. whether it is due to increased perceived risk, distraction, stress or masking) needs targeted experiments [11,51].

The similar vigilance level exhibited during road-noise playback, whether it was preceded by close-call or alarm-call playback, suggests no additive or synergistic effect of the two stressors. There is the possibility of an antagonistic effect because the vigilance seen in the dual-stressor treatment was less than the sum of the effects from the two stressors alone [19]. However, some caution with that interpretation is required because the mongoose response to an alarm call was relatively brief, and there was a 1 min gap between the individual hearing the alarm call before they were exposed to the road noise. The lack of a vigilance difference between the two treatments that finished with ambient-sound playback suggests that the greater vigilance resulting from the alarm call had finished by the time the second playback (of ambient sound or road noise) occurred. A recent experiment with mussels (*Mytilus* spp.) found no combined effect of elevated predation risk and noise [52]. In contrast, the reduction in food intake of small mammals in response to an elevated predation risk was eliminated by road-noise playback [28]. In that latter study, the dual stressors were presented simultaneously, whilst they were sequential in our experiment. We also exposed the dwarf mongooses to the stressors for very short periods. In contrast, in a study of great tits (*Parus major*), road noise was played continuously for several days in the treatment week [23]. Changes in response might be expected with repeated or chronic exposure [53], although the mongooses did respond to the road-noise playback in isolation. Finally, most studies looking at the effects of combined stressors on animals have focused on light and noise pollution [22–25]. An additive or synergistic effect might be more likely if the two stressors are both man-made—animals have had less time to adapt to those than to natural stressors. In principle, such a cumulative effect might be found if the stressors that we simulated occurred in the opposite order. But, future work would be needed to assess these possibilities.

Our results show that both an elevated predation risk and road noise cause individuals to be more vigilant; future work could investigate whether there are inter-individual differences in response due to, for example, sex, age, dominance status, proximity of groupmates or group size [21]. For many species, including dwarf mongooses, increased vigilance is traded off against other important activities such as foraging. Care is needed when extrapolating from short-term experiments, because chronic exposure can lead to reduced responses over time, and there is the possibility for compensatory behaviour in less-stressful periods, but there is increasing evidence that anthropogenic noise can have fitness consequences [5,54]. Determining the full impacts of noise pollution, and thus developing suitable mitigation strategies, will therefore require more research into longer-term and combined-stressor effects.

## Data Availability

Data and code required to replicate all analyses are available in the electronic supplementary material files (‘Call_playback_responses’, ‘Road_playback_responses’ and ‘Mongoose_dual_stressors_data_code’). All information about how the data were collected and processed is contained within the main paper and the electronic supplementary material. Supplementary material is available online [55].
